# A planning strategy may reduce the risk of heart diseases and radiation pneumonia: Avoiding the specific heart substructures

**DOI:** 10.1002/acm2.14119

**Published:** 2023-08-11

**Authors:** AiHui Feng, YanHua Duan, ZhangRu Yang, Yan Shao, Hao Wang, Hua Chen, HengLe Gu, Ying Huang, ZhenJiong Shen, Xufei Wang, ZhiYong Xu

**Affiliations:** ^1^ Institute of Modern Physics Fudan University Shanghai China; ^2^ Key Laboratory of Nuclear Physics and Ion‐beam Application (MOE) Fudan University Shanghai China; ^3^ Department of Radiation Oncology Shanghai Chest Hospital School of Medicine Shanghai Jiao Tong University Shanghai China

**Keywords:** avoidance plan, cardiac substructures, heart toxicity, lung cancer

## Abstract

**Background:**

Dose to heart substructures is a better predictor for major adverse cardiac events (MACE) than mean heart dose (MHD). We propose an avoidance planning strategy for important cardiac substructures.

**Material and methods:**

Two plans, clinical and cardiac substructure‐avoidance plan, were generated for twenty patients. Five dose‐sensitive substructures, including left ventricle, pulmonary artery, left anterior descending branch, left circumflex branch and the coronary artery were chosen. The avoidance plan aims to meet the target criteria and organ‐at‐risk (OARs) constraints while minimizing the dose parameters of the above five substructures. The dosimetric assessments included the mean dose and the maximum dose of cardiac substructures and several volume parameters. In addition, we also evaluated the relative risk of coronary artery disease (CAD), chronic heart failure (CHF), and radiation pneumonia (RP).

**Results:**

Pearson correlation coefficient and *R* ^2^ value of linear regression fitting demonstrated that MHD had poor prediction ability for the mean dose of the cardiac substructures. Compared to clinical plans, an avoidance plan is able to statistically significantly decrease the dose to key substructures. Meanwhile, the dose to OARs and the coverage of the target are comparable in the two plans. In addition, it can be observed that the avoidance plan statistically decreases the relative risks of CAD, CHF, and RP.

**Conclusions:**

The substructure‐avoidance planning strategy that incorporates the cardiac substructures into optimization process, can protect the important heart substructures, such as left ventricle, left anterior descending branch and pulmonary artery, achieving the substantive sparing of dose‐sensitive cardiac structures, and have the potential to decrease the relative risks of CAD, CHF, and RP.

## 1 INTRODUCTION

During radiotherapy of patients with locally advanced non‐small cell lung cancer (NSCLC), the heart may receive a relatively high radiation dose, inducing a higher risk of adverse cardiac events.^1^, ^2^, ^3^, ^4^, ^5^ The dose to arterial blood vessels will increase the risk to induce inflammation, accelerating the development of coronary disease.^6^, ^7^ Radiotherapy can also damage cardiomyocytes, resulting in abnormal myocardial structure and dysfunction, and eventually leading to heart failure or cardiac death. The consequences of cardiac events in patients with NSCLC are associated with cardiac radiation dose and baseline cardiac risk.

Research shows that the increased risk of radiation‐induced heart disease (RIHD), including acute (pericarditis) and late (congestive heart failure, coronary artery disease [CAD], and myocardial infarction) cardiotoxicity depends on many factors, including the RT prescription,^8^ patients’ baseline cardiac risk,^3^ as well as the planning optimization process.^9^ The occurrence of RIHD is earlier than previously expected, not only a few years after RT, but the risk of heart events will last for around 20 years. With the development of new technologies, such as immunotherapy^10^ and targeted therapy, the survival time of lung cancer patients prolongs, and risk of heart disease also increases.

Previous studies^8^, ^11^, ^12^ have evaluated the radiation exposure of the heart by the mean heart dose (MHD). However, MHD has been shown to be a poor surrogate parameter for the dose of coronary artery.^13^ Studies have shown that the dose of heart substructure is not only related to the overall survival (OS) and cardiotoxicity but also related to the pulmonary toxicity. Atkins's^14^ cohort study demonstrated that the left anterior descending branch (LAD) V_15Gy_ greater than or equal to 10% appeared to be an independent estimator of the probability of major adverse cardiac Events (MACE) and all‐cause mortality. Jacob et al.^13^ illustrated that MHD was not enough to predict with confidence individual patient dose to the left ventricle (LV) and coronary arteries. McWilliam^15^ investigated the data of 1161 patients and it was confirmed that the maximum dose to the combined cardiac region encompassing the right atrium, right coronary artery, and ascending aorta was found to have the greatest effect on patient survival. In addition, Tomita^16^ found that pulmonary artery (PA) V_35Gy_ is related to the development of radiation pneumonia (RP), and the moderate dose sparing of PA could reduce the risk of RP in thoracic radiotherapy. Different heart substructures are likely to have different dose–risk relationships. Therefore, radiotherapy cardiotoxicity risk assessment model based on MHD may be misleading. Therefore, it is very important to delineate the cardiac substructure and evaluate their cardiotoxicities. Levis^9^ reduced the risk of cardiovascular events through an optimization of the dose distribution on heart substructures in mediastinal Hodgkin lymphoma (HL) patients. Based on their research, we conducted radiotherapy planning strategy to spare the dose‐sensitive cardiac substructures for patients with NSCLC. By adopting advanced risk estimation formula, the dosimetric gain was successfully translated in a reduced risk of CAD, chronic heart failure (CHF), and PR. We also added a moderate dose sparing of PA to reduce the risk of RP in thoracic radiotherapy.

In a group of patients receiving conventional radiotherapy, the substructure‐avoidance planning strategy was compared with the previous clinical plans (using whole heart as the constraint objective). In addition, the possibility of risk reduction of CAD, CHF, and RP was investigated by the risk prediction model, providing more intuitive biological data support for the substructure‐avoidance plan.

## 2 MATERIAL AND METHODS

### 2.1 Patients selection

Twenty patients with NSCLC who received radiotherapy in Shanghai Chest Hospital from January to June 2021 were selected. We sought to maintain a population of patients who received similar doses and had similar disease burden nearby the central thoracic structure. Therefore, we included patients with stage III‐IV disease in this research. These patients were expected to have a relatively bulky burden of central thoracic disease, so these cases had similar requirements in target coverage and as well as the constraint of organ‐at‐risks (OARs). Additionally, we only included patients who received 60 Gy, 2 Gy per fraction to exclude the conversion of the biologically effective dose and the equivalent dose to 2 Gy fractions. Detailed patient characteristics are shown in Table 1.

**TABLE 1:** Tumor and treatment characteristics.

Characteristic	Number	%
Patients	20	100
Age (years)		
Range	20‐88	
Median	63	
Sex		
Male	16	80
Female	4	20
Stage		
IIIa	6	30
IIIb	10	50
IV	4	20

### 2.2 Contour delineations

One radiation oncologist performed the delineations of the clinical target volume (CTV) and OARs for all patients. Considering the use of daily cone beam computed tomography (CBCT) image guidance, an 8‐mm isotropic margin was added to the CTV to generate the planning target volume (PTV). Cardiac substructures, including the aortic arch, the coronary artery (left main artery [LMA], left anterior descending artery [LAD], left circumflex [LCX], and right coronary artery [RCA]), the chambers (left atrium [LA], right atrium [RA], left ventricle [LV]. and right ventricle [RV]), ascending aorta (AA), descending aorta (DA), superior vena cava (SVC), inferior vena cava (IVC), pulmonary artery (PA), pulmonary vein (PV), and the whole heart, were manually delineated on archived planning CT scans in MIM Maestro, version 7.1.4 (MIM Software Inc, Cleveland, OH). CT scans were acquired without intravenous contrast. According to the published atlas of Feng,^17^ which provides a detailed description of cardiac anatomy, the whole heart and the above‐mentioned heart substructures were contoured by a radiation oncologist. Since the pericardium cannot be identified on CT images, we used a surrogate pericardium (PC), by creating a 3‐dimensional structure with the WH contour as inner border and the WH + 5 mm as outer border. The adoption of contour guidelines for cardiac substructures can make up for the omission of intravenous contrast injection. In fact, Feng^17^ noted that intravenous contrast did not improve the accuracy of contours or radiation dose reported when atlas was properly applied. Afterwards, another radiation oncologist independently reviewed the cardiac substructures.

### 2.3 Radiotherapy technique

For each patient, two plans were generated: substructure‐avoidance and clinical plan. The clinical plan adopts the target and OAR optimization setting specified by our center, in which the dose constraints of the heart is for the whole heart. The substructure‐avoidance plan is aimed at sparing the cardiac substructure to the greatest extent, while maintaining target coverage and meeting the standard limits for OARs.

According to previous research, specific margins for coronary artery were used to compensate the displacement related to the cardiac motion. The cardiac motion is evaluated by using ECG gated CT scanning.^18^ In short, the isotropic margins of OARs by applying McKenzie—van Herk formula, were: 5 mm for LAD and RCA, 4   mm for LCX, and 3 mm for LMA. The motions of LA, RA, LV, and RV were not investigated before, so the same margins of the coronary artery were used in this planning process.

The plans were created on Pinnacle^3™^ treatment planning system (TPS, v9.10, Philips Medical Systems, Cleveland) with auto‐planning module. All OARs including whole heart (WH) and PTV contours were taken from the original clinical plans. IMRT with five to eight coplanar 6‐MV flat beams was used for the patients reported in this paper. The beam directions were customized for individual patients. Intensity modulation was performed using the direct machine parameter optimization (DMPO) algorithm, where the maximum number of multi‐leaf collimator (MLC) segments was set at ten. For each plan, dose distributions were calculated using the collapsed cone convolution algorithm (CCC) with a calculation grid of 3×3×3 mm^3^. Detailed optimization objectives and priorities are listed in Table 2.

**TABLE 2:** Starting optimization objectives and weights applied during clinical plan optimization (CL) and avoidance plan optimization (AV).

Structure	Planning	Type	Dose(cGy)	Volume (%)	Priority	Comprise
Total Lung	CL	Max DVH	500	40	medium	Yes
	CL	Max DVH	2000	20	medium	Yes
	CL	Max DVH	3000	15	medium	Yes
Spinal Cord+2 mm expansion	ALL	Max dose	4400	/	high	No
Spinal Cord+5 mm expansion	ALL	Max dose	4500	/	high	No
Heart	ALL	Max DVH	3000	25	high	Yes
	ALL	Max DVH	4000	35	high	Yes
LV+5 mm expansion	AV	Max DVH^29^	500	ALARA	high	Yes
	AV	Max DVH^14^	1500	1	high	Yes
PA+5 mm expansion	AV	Max DVH^16^	3500	ALARA	high	Yes
LAD+5 mm expansion	AV	Max DVH^14^	1500	10	high	Yes
	AV	Max Dose^30^	10 Gy	/	high	Yes
LCX+4 mm expansion	AV	Max DVH^14^	1500	14	high	Yes
Coronary sum	AV	Mean^14^	700	/	high	Yes

*Note*: In case the objective is used in both planning techniques, it is labeled as ALL. If the objective is used merely in clinical plan or avoidance plan, it is labeled as CL or AV, respectively. The “ALARA” means that the dose to cardiac substructures were reduced according to as low as reasonably achievable (ALARA) principles.

### 2.4 Risk estimation and statistical analysis

The CAD prediction adopts the HL survivor risk assessment model published by van Nimwegen.^19^ The prediction model demonstrated a linear dose‐response relationship between MHD and the risk of CAD. The relative risk (ERR) of coronary events was 7.4%/Gy. Darby^20^ first observed this model in patients with breast cancer. We imitated the research of Levis and replaced MHD with the mean dose of coronary artery to calculate the risk of CAD.

For the estimation of CHF, we adopted the risk model of van Nimwegen,^21^ which showed the linear dose‐response relationship between mean dose of the left ventricular and the CHF risk, with an ERR for clinical events of 9.0% per Gy.

For the calculation of RP risk, the Cox single factor regression model proposed by Tomita^16^ is adopted, which considers the HR ratio of PA V_35Gy_ to RP ≥grade 2 is 1.04.

Based on the above models, the risks of CAD, CHF and RP were calculated and evaluated. Afterwards we compared the dosimetric parameters of the clinical plan and the substructure‐avoidance plan, as well as the CAD, CHF and PR. The main parameters we calculated were found to have nonnormal distributions and were evaluated by Wilcoxon rank test. The D_max_ (which we use D_0.03cc_ instead) and the mean dose (D_mean_) to OARs, and the lung volume receiving 5 Gy(V_5Gy_), 20 Gy(V_20Gy_) were collected.

Pearson correlation coefficient was used to analyze the correlation between MHD and the mean dose to cardiac substructures. In addition, the relationship between MHD and the mean dose to substructures were also analyzed based on linear regression, and the *R* ^2^ value was calculated. *R* ^2^ < 0.70 was considered that the MHD was not enough to predict and replace the dose parameters of substructures. *p* < 0.05 was considered statistically significant. All statistical analyses were performed by using SPSS (version 12.0; SPSS Inc., Chicago, IL).

## 3 RESULT

### 3.1 The association between MHD and the mean dose to substructures

Pearson correlation coefficients between the MHD and the mean dose to cardiac substructures were listed in Table 3. Additionally, the relationship analysis between MHD and mean doses to the different substructures were further investigated based on linear regressions providing the *R* ^2^ value, which were also listed in Table 3.

**TABLE 3:** Association parameter between the mean dose to substructures and MHD.

Structure	Pearson correlation coefficient	*p*‐value	*R* ^2^
DA	0.30	0.202	0.04
AA	−0.37	0.112	0.09
PA	0.15	0.53	0.02
SVC	**−0.59**	0.007	0.31
PV	0.24	0.319	0.00
Aortic Arch	**−0.59**	0.007	0.31
LV	**0.69**	0.001	0.44
LA	0.40	0.079	0.12
RV	**0.81**	<0.001	0.63
RA	−0.14	0.552	0.02
IVC	0.21	0.385	0.04
Pericardium	0.32	0.175	0.10
LMA	**0.45**	0.047	0.16
LAD	**0.46**	0.04	0.17
LCA	0.44	0.055	0.14
RCA	−0.17	0.475	0.03

*Note*: The Pearson correlation coefficient with statistical significance is displayed in bold.

Portions of SVC and Aortic Arch are not included in the heart delineation, so it is understandable that the mean dose of them has a medium negative correlation with MHD. RV was observed to be strongly correlated with MHD (*r* = 0.81), followed by LV (*r* = 0.69), and LMA (*r* = 0.45) and LAD (*r* = 0.46) were moderately correlated. The dose difference of other substructures was not statistically significant.

**FIGURE 1:**
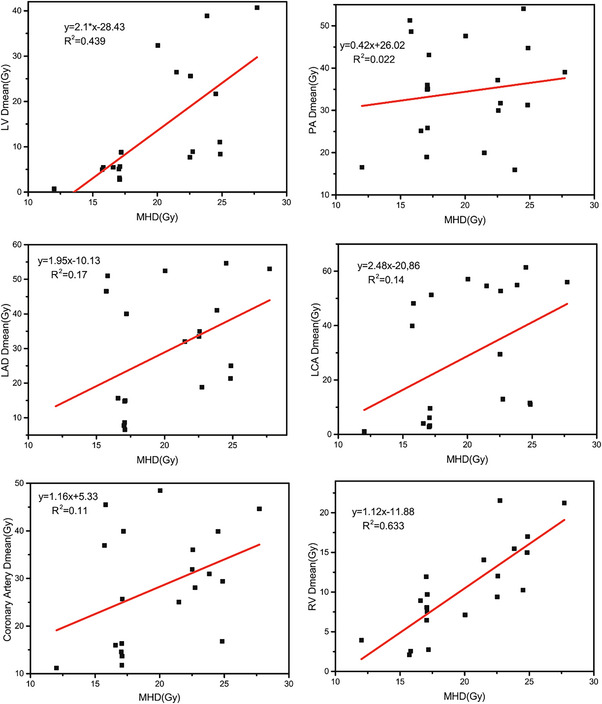
The association between D_mean_ of substructures and MHD.

Linear regression between MHD and D_mean_ for cardiac substructures are presented in Figure 1. For every increase in MHD, the D_mean_ LV increased 2.1 Gy, the D_mean_ PA increased 0.42 Gy, the D_mean_ LAD increased 1.95 Gy, the D_mean_ LCA increased 2.85 Gy and the coronary artery increased 1.16 Gy. However, the correlation coefficient of determination *R* ^2^ values indicated that, the D_mean_ RV predictable from MHD was moderate (*R* ^2^ = 0.66), and low for D_mean_ LV(*R* ^2^ = 0.44), while for D_mean_ PA, D_mean_ LAD, D_mean_ LCA and coronary artery, there is almost no predictive ability from MHD (*R* ^2^ < 0.2). It can be inferred that MHD has poor predictive ability for D_mean_ of substructure with no *R* ^2^ value above 0.70. Therefore, it is necessary to carry out substructure‐avoidance plan for specific structure.

### 3.2 Clinical and substructure‐avoidance plans for example patient

**FIGURE 2:**
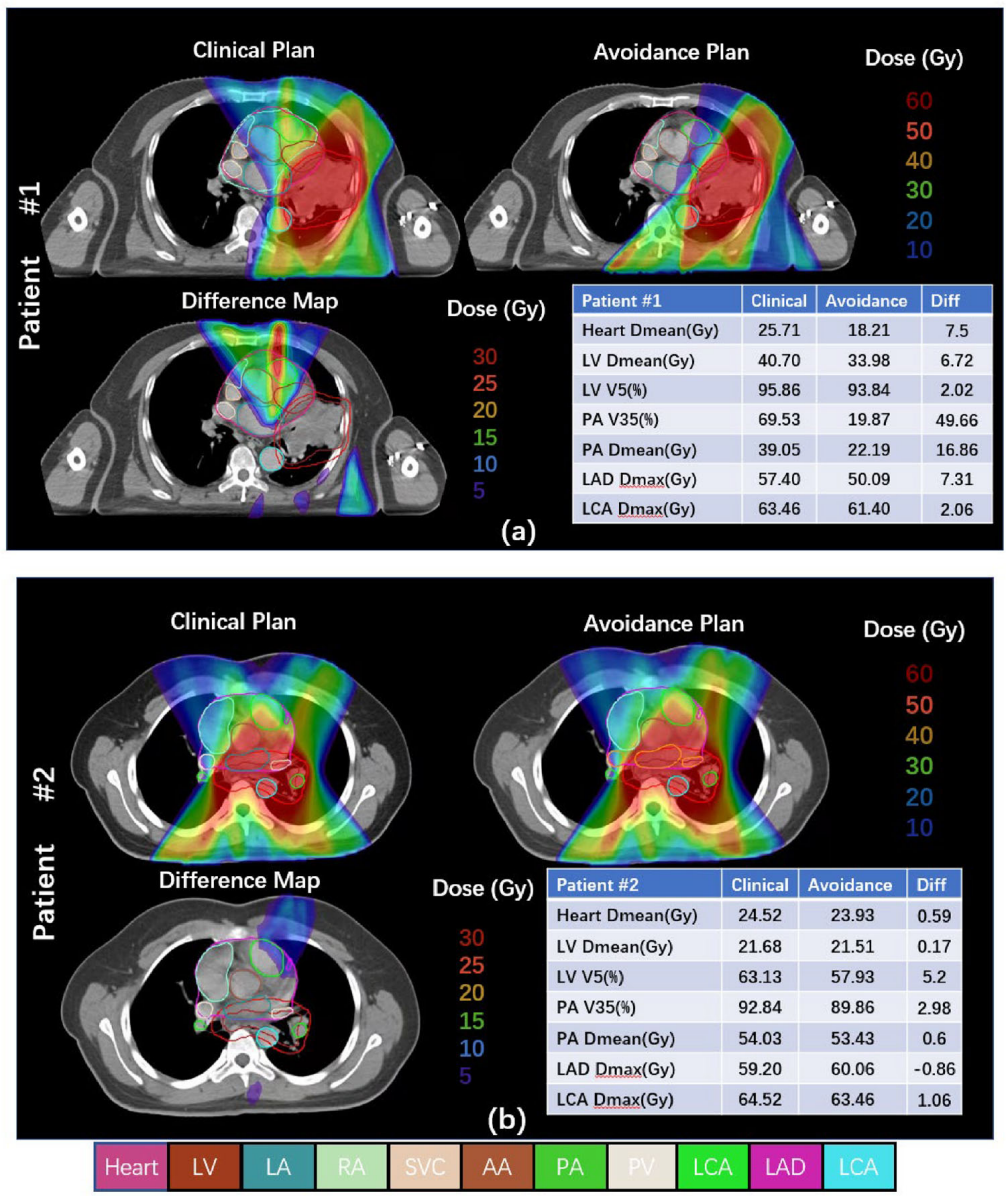
Example isodose distributions and difference map of clinical and heart substructure‐avoidance plans of two patients. Dose metric table shows standard whole heart dose metrics and substructure metrics. Difference maps are the dose of clinical plan minus the avoidance plan.

Figure 2 shows the dose distribution and dose metrics of the clinical and substructure‐avoidance plan for two patients. The dose distribution of patient 1 demonstrates that the substructure‐avoidance plan can spare the LV, LAD and PA, decreasing the low‐dose bath in cardiac substructures. As shown in Figure 2a, the D_mean_ of LV, LAD and PA, as well as the V_35Gy_ of PA decreased significantly. For patient 2, there is no large difference between the dose distribution or dose metrics of the two plans, as we can see in the dose difference map in Figure 2b. This is due to the fact that the target area overlaps with the substructures such as PA, so it is hard to carry out beam avoidance and dose sparing for these substructures. Therefore, the substructure‐avoidance plan also needs individual design, taking the patient's anatomical structure and target location into account comprehensively.

### 3.3 Comparison of heart substructure metrics between clinical and avoidance plans

Table 4 presents the D_max_ and D_mean_ of the whole heart and cardiac substructures, as well as important volume parameters. While the PTV and other OARs meet the clinical constraints (Table 5), the dose metrics of the heart in the avoidance plan have decreased statistically significantly. Compared to the clinical plan, we added the optimization objective for other substructures in the avoidance plan, including the LV, LAD, LCX, PA, and the coronary artery. All of the above substructures decreased statistically significantly. Among them, the LV V_5Gy_ dropped from 49.31% to 38.77%, the PA V_35Gy_ dropped from 49.71% to 40.49%, the LAD V_15Gy_ dropped by 20.51%, and the mean dose of the coronary artery dropped from 28.14 Gy to 21.45 Gy. Meanwhile, the mean dose ​​of DA, AA, PV, RV, RA, IVC, LM, LCA in the avoidance plan also decreased compared to the clinical plan, and the decreases in other substructures except for RA and IVC are statistically significant. Of course, we have also noticed that the D_mean_ of several substructures has increased, which is, SVC and AA. These two structures are not included by the heart contour, and are much closer to the PA which we set as the optimization constraints, resulting in the additional dose deposition on SVC and AA.

**TABLE 4:** Dose parameters of heart substructures for 20 patients with clinical and avoidance plan.

Parameter		Clinical	Avoidance	*p*‐value
Heart	V_30Gy_ (%)	27.31 ± 7.01	22.22 ± 6.28	<0.001
	V_40Gy_ (%)	19.04 ± 5.45	15.38 ± 5.25	0.002
	MHD (Gy)	19.89 ± 4.14	17.35 ± 3.60	<0.001
Pericardium	D_max_ (Gy)	65.74 ± 0.93	66.40 ± 0.85	0.004
	D_mean_ (Gy)	23.18 ± 3.13	21.22 ± 3.85	<0.001
DA	D_max_ (Gy)	55.47 ± 14.96	56.58 ± 14.19	0.005
	D_mean_ (Gy)	23.02 ± 13.60	22.41 ± 14.05	0.017
AA	D_max_ (Gy)	60.49 ± 8.89	58.10 ± 15.24	0.823
	D_mean_ (Gy)	39.39 ± 12.33	37.46 ± 14.34	0.04
PA	D_max_ (Gy)	65.18 ± 1.08	65.85 ± 0.87	0.004
	D_mean_ (Gy)	34.35 ± 11.61	31.05 ± 12.83	<0.001
	V_35Gy_ (%)	49.72 ± 26.69	40.49 ± 27.01	<0.001
SVC	D_max_ (Gy)	54.21 ± 21.27	53.97 ± 22.04	0.575
	D_mean_ (Gy)	37.33 ± 20.89	37.43 ± 21.27	0.167
PV	D_max_ (Gy)	64.27 ± 1.56	65.01 ± 1.16	0.004
	D_mean_ (Gy)	33.71 ± 13.53	32.09 ± 14.05	0.001
Aortic Arch	D_max_ (Gy)	44.34 ± 27.12	44.67 ± 27.37	0.179
	D_mean_ (Gy)	19.06 ± 18.31	19.14 ± 18.74	0.911
LV	D_max_ (Gy)	48.28 ± 14.89	42.74 ± 18.63	0.006
	V_5Gy_ (%)	49.31 ± 31.51	38.77 ± 31.97	<0.001
	V_15 Gy_ (%)	27.41 ± 30.75	21.99 ± 29.33	<0.001
	V_23 Gy_ (%)	20.17 ± 27.09	14.71 ± 21.65	<0.001
	D_mean_ (Gy)	13.33 ± 12.70	9.94 ± 10.49	<0.001
LA	D_max_ (Gy)	64.22 ± 1.41	64.58 ± 1.93	0.167
	D_mean_ (Gy)	26.79 ± 8.46	24.65 ± 1.93	0.001
	V_10Gy_ (%)	69.21 ± 20.36	62.59 ± 19.71	0.005
RV	D_max_ (Gy)	38.73 ± 12.77	31.66 ± 13.67	0.003
	D_mean_ (Gy)	10.36 ± 5.73	8.00 ± 4.37	<0.001
RA	D_max_ (Gy)	50.92 ± 16.24	48.91 ± 20.21	0.765
	D_mean_ (Gy)	25.12 ± 15.55	24.08 ± 15.64	0.391
IVC	D_max_ (Gy)	16.14 ± 19.85	16.31 ± 19.71	0.97
	D_mean_ (Gy)	8.70 ± 12.92	8.40 ± 12.08	0.709
LM	D_max_ (Gy)	42.52 ± 17.50	37.72 ± 18.95	0.002
	D_mean_ (Gy)	36.15 ± 17.31	28.90 ± 18.66	<0.001
LAD	V_15Gy_ (%)	68.92 ± 40.94	48.41 ± 47.48	0.003
	D_max_ (Gy)	35.20 ± 19.10	27.81 ± 20.79	<0.001
	D_mean_ (Gy)	28.70 ± 17.49	20.58 ± 16.86	<0.001
LCA	V_15Gy_ (%)	53.50 ± 45.01	46.35 ± 48.65	0.028
	D_max_ (Gy)	36.69 ± 24.24	32.42 ± 26.35	0.001
	D_mean_ (Gy)	28.54 ± 23.68	23.47 ± 21.82	<0.001
RCA	D_max_ (Gy)	31.19 ± 13.40	25.60 ± 12.76	0.004
	D_mean_ (Gy)	25.22 ± 12.29	18.36 ± 9.75	<0.001
Coronary artery	D_max_ (Gy)	51.88 ± 11.96	47.92 ± 15.58	0.01
	D_mean_ (Gy)	28.14 ± 12.15	21.45 ± 12.30	<0.001

**TABLE 5:** Critical organ and PTV metrics of clinical and avoidance plans for 20 lung cancer patients.

Parameter		Clinical	Avoidance	*p*‐value
Heart	V_30Gy_ (%)	27.31 ± 7.01	22.22 ± 6.28	<0.001
	V_40Gy_ (%)	19.04 ± 5.45	15.38 ± 5.25	0.002
	MHD (Gy)	19.89 ± 4.14	17.35 ± 3.60	<0.001
Total Lung	V_5Gy_ (%)	38.47 ± 6.80	38.54 ± 6.62	0.526
	V_20Gy_ (%)	20.77 ± 4.48	21.04 ± 4.72	0.052
	MLD (Gy)	11.59 ± 2.16	11.65 ± 2.17	0.455
Spinal Cord	D_max_ (Gy)	44.65 ± 3.15	45.75 ± 2.85	0.001
PTV	D2 (Gy)	64.78 ± 0.56	65.33 ± 0.62	0.001
	D50 (Gy)	62.68 ± 0.38	63.07 ± 0.36	0.001
	D98 (Gy)	58.93 ± 0.45	58.57 ± 0.53	0.001
	CI	0.73 ± 0.06	0.66 ± 0.08	<0.001
	HI	9.32 ± 1.46	10.70 ± 1.57	<0.001
	Monitor units	507 ± 140	506 ± 145	0.881
	Number of segments	30 ± 12	28 ± 13	0.477

In addition, the D_max_ of some cardiac substructures has also decrease due to the newly added optimization settings. For example, the LV D_max_ decreased from 48.28 Gy to 42.74 Gy (*p* < 0.05). There were also statistically significant increases in the D_max_ of cardiac substructures, including pericardium, DA, PA, and PV. However, the increase of these structures is small, with the maximum value of 1.11 Gy for DA. No clinical significances have been raised by these substructures, and further investigation as well as long term follow‐up are needed.

### 3.4 The comparison of PTV and other OARs between clinical and avoidance plans

Comparing to the clinical plan, D2 increased statistically significantly, and D98 decreased statistically significantly (*p* < 0.05) in substructure‐avoidance plan. The conformation index (CI) of PTV decreased from 0.73 ± 0.06 in the clinical plan to 0.66 ± 0.08 in the avoidance plan (*p* < 0.001), with a relative decrease of 9.59%. Moreover, in the avoidance plan, the heterogeneity index (HI, defined as (D2‐D98)/D50) also increased from 9.32 ± 1.46 to 10.70 ± 1.57 (*p* < 0.001). Compared to the clinical plan, the MU of the avoidance plan increased (507 ± 140 vs. 506 ± 145), and the number of beam segments also increased (30 ± 12 vs. 28 ± 13), yet no statistically significant differences were observed.

Table 5 lists the dose metrics of the total lung, spinal cord, and heart for 20 patients. The dose parameters of whole heart (V_30Gy_, V_40Gy_ and MHD) in the avoidance plan are smaller than those in the clinical plan, with statistically significantly difference. For the total lung, the dose metrics of the avoidance plan are slightly higher than that in the clinical plan, but the absolute difference is quite small and are not statistically significant (*p* > 0.05). For the spinal cord, the D_max_ in the avoidance plan is larger than the clinical plan, yet all dose metrics in the avoidance plans meet the dose constraints set by our center.

### 3.5 The prediction of the relative risk of CAD, CHD, and PR

**FIGURE 3:**
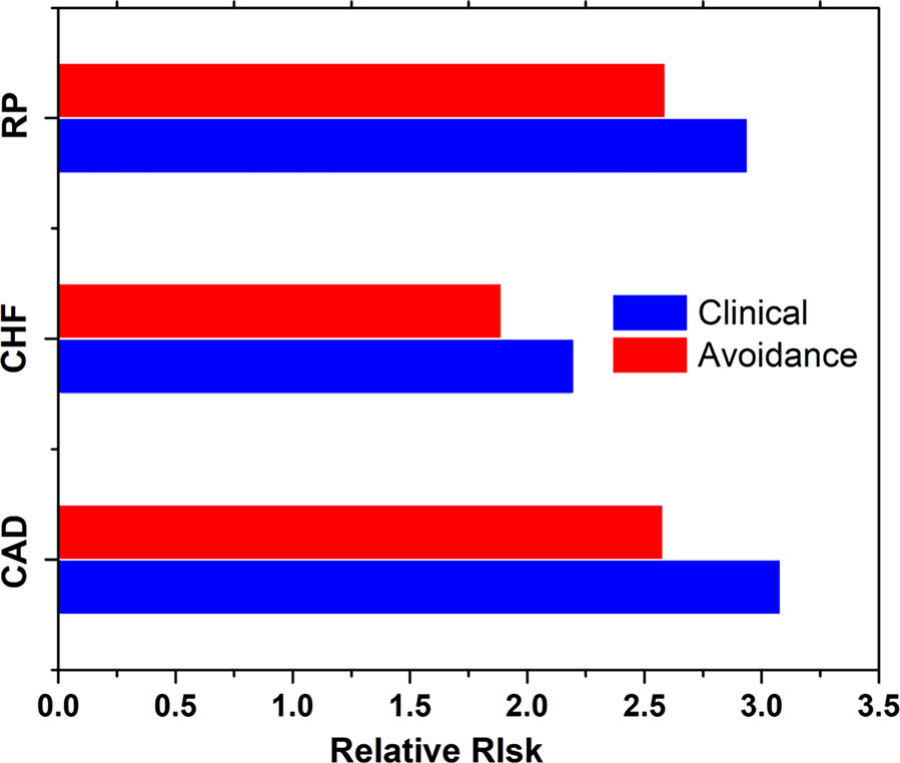
The relative risks of CAD, CHF, and RP for the two plans.

Figure 3 and Table 6 present the excess relative risks (ERR, %) of CAD, CHF and PR in clinical plans and avoidance plans. The dose gains in Coronary artery, LV and PA induce in lower relative risk (RR) for CAD (3.08 vs. 2.58, *p* < 0.001), CHF (2.2 vs. 1.89, *p* < 0.001), and RP (2.94 vs. 2.59, *p* < 0.001), respectively.

**TABLE 6:** The relative risks of CAD, CHF, and RP for the clinical and avoidance plan.

Target	Mean value (± SD)		
	Clinical plan	Avoidance plan	*p*‐value
CAD	3.08 ± 0.90	2.58 ± 0.91	<0.001
CHF	2.20 ± 1.14	1.89 ± 0.94	<0.001
RP	2.94 ± 1.05	2.59 ± 1.06	<0.001

## 4 DISCUSSION

As the development of radiotherapy technology, more patients with lung cancer whose survival time is extended are plagued by MACE and radiation pneumonitis, resulting in the decrease in quality of life. Researchers have demonstrated the fact that MHD is not enough to predict with confidence individual patient dose to the cardiac substructures. The sole constraint to the whole heart cannot effectively reduce the incidence of CAD, CHF, and RP since it is unable to account for the variability and steepness of dose distributions across cardiac regions. Our study showed that MHD is indeed insufficient to predict the mean dose to substructures. For this reason, a new strategy of radiotherapy planning for evasive protection of dose‐sensitive cardiac substructures has been proposed, which can make up for the shortcomings of MHD's insufficient predictable ability and has the potential to lower the risks of CAD, CHF, and RP.

One strength of our study is that two planning process were conducted on planning organs at risk volume (PRVs), the application of specific margins to the coronary arteries, left and right ventricles, and atria to calculate the dose of these structures much more accurately. Moreover, we have proposed dedicated planning objectives for specific cardiac substructures (Table 2), with explicit clinical data support. In addition, we are the first team to incorporate PA sparing into radiation therapy planning which may reduce the risk of RP. Finally, a more intuitive biological data support for the substructure‐avoidance plan is also the strength of this paper. We have taken a step towards the detailed planning of dose spatial distribution and the management of adverse cardiac reactions in patients with lung cancer caused by radiotherapy.

The Pearson correlation coefficient between MHD and D_mean_ to cardiac substructure listed in Table 3 shows the facts that except for RV, LV, LM, and LAD structures are moderately correlated with MHD, and other structures even do not have correlation (*r* < 0.2). The linear regression results in Figure 1 also show that MHD has insufficient prediction ability for substructure. The results of Jacob^13^ and Hoppe^22^ are consistent with our data. Since the fact that MHD can not accurately show the dose delivered to different anatomical structures, our spatially localized dose calculation is of special value to lung cancer patients who may have adverse cardiac events and RP.

At present, few studies have integrated cardiac substructure into the optimization process of treatment planning. Levis et al.^9^ have shown the importance of cardiac substructure. When evaluating the treatment regimen of B‐VMAT and FaB‐VMAT, they made a dosimetric comparison on the cardiac substructure of coronary artery. And a significant decrease in coronary artery was obtained by changing the direction of radiation beams. Morris^23^ introduced 0.35T MRIs to verify cardiac substructure segmentations for CT‐based treatment planning, designed the cardiac substructure sparing plan for patients with upper chest cancers, without involving the relationship between dose gain and cardiotoxicity. Based on their research, we investigated the dose constraints of LV, PA, LAD, LCX and the coronary artery. Our novel planning strategy significantly decrease the dose to the extremely important substructure proposed by clinical studies,^14^, ^16^, ^18^ including V5_Gy_ andV_15Gy_ of the LV, V_35Gy_ of the PA, V_15Gy_ of the LAD, V_15Gy_ of the LCX and mean dose of the coronary artery. Meanwhile the avoidance plan achieves comparable target coverage and the dose to the total lung to the clinical plan, even though the spinal cord D_max_ increased slightly (1.1 Gy). And the above metrics were still within the clinical limits. In addition, a significant difference in MHD among the two planning processes (2.5 Gy less with the “avoidance” optimization) was found. This is due to the avoidance of the specific substructures lead to the protection of surrounding substructures, as we can see in Table 4. Compared with the clinical plan, the difference in MU and beam segments of avoidance plan were negligible (*p* > 0.05) (Table 5), which confirmed that the complexity of the two plans was quite similar. This demonstrated that the actual burden of accelerator was also merely affected by the integration of cardiac substructures into the planning process.

At the same time, we calculated the excess relative risks of CAD, CHF and RP, authenticating the toxicity strengths of the cardiac substructure‐avoidance strategy. Different cardiac substructures differ in dose‐risk relationships, hence we used three kinds of models in toxicity analysis to further verify the clinical gains of the avoidance plan. Of course, although the heart substructure has been accurately delineated and specific risk model has been applied, there are many other issues of heart events, and the existing biological model has not been fully established. Therefore, we have not studied the non‐radiation factors, such as the use of anthracycline^24^ and the associated cardiovascular risk factors (for instance, hypertension, diabetes, dyslipidemia and obesity).^19^, ^20^ Establishing a complete biological model may be the future research direction.

In our study, the manual delineations of cardiac substructure were time‐consuming and had an expensive time cost in clinical applications. The delineations of heart substructures were conducted on a non‐contrast CT, just as the study of Levis^9^ and Morris.^23^ The dosimetry strategies of re‐optimization using cardiac substructures are applicable to other X‐ray‐based treatment planning modalities as our atlas and manual substructure segmentations work on CT image inputs. Altogether, the results presented in this work are applicable to a variety of settings, tumor sites, breathing states, and fractionation schedules, which appear promising for future work in cardiac sparing. However, some studies have discussed the atlas‐based or automatic segmentation methods for cardiac substructures,^25^, ^26^ which is helpful for efficiently standardizing the contouring of the cardiac substructures. In addition, for few patients, due to the fact that the target overlaps with key cardiac substructures, or the tumor is quite large, as shown in Figure 2 patient 2. If it is not possible to reduce the dose exposure of the cardiac substructure to an ideal level without affecting the target coverage, other treatment options can be considered, such as proton radiation^27^ or non‐radiation treatment.^28^

## 5 CONCLUSION

This study proposes a novel avoidance planning strategy for cardiac substructure with the comparable target coverage, OAR dose and plan complexity to the clinical plan. And significantly dose decreases in key substructures (LV, PA, LAD, LCX and coronary artery) were observed. This work shows the strong potential of cardiac substructure‐avoidance plans in reducing the risk of cardiac adverse events and RP. Therefore, we recommend that cardiac substructures be routinely included in the optimization of NSCLC cases as a basis for improving OAR protection, especially as an effective method for cardiac dosimetry.

## AUTHOR CONTRIBUTIONS

AiHui Feng was involved in conceptualization, investigation, methodology, software, data curation, writing original draft preparation, and review/editing. YanHua Duan was involved in conceptualization, methodology and review/editing. ZhangRu Yang was involved in data curation, writing review, and editing. Yan Shao was involved in conceptualization, methodology, and software. Hao Wang was involved in data curation and visualization. Hua Chen was involved in software, writing review, and editing. HengLe Gu was involved in software. Ying Huang was involved in software and formal analysis. ZhenJiong Shen was involved in Data curation and formal analysis. XuFei Wang was involved in conceptualization, methodology, project administration, supervision, funding acquisition, and review/editing. ZhiYong Xu was involved in conceptualization, methodology, project administration, supervision, and review/editing. All authors contributed to the article and approved the submitted version.

## CONFLICT OF INTEREST STATEMENT

The authors declare no conflicts of interest.
